# Synergistic antibacterial activity and mechanism of arginine combined with florfenicol against *Escherichia coli*

**DOI:** 10.1080/21505594.2026.2666994

**Published:** 2026-05-04

**Authors:** Mingyu Cao, Yuhang Li, Guangfu Han, Hanyu Liu, Shanshan Xiong, Xiaona Liu, Menghan Zhao, Yuefan Su, Yanhua Li, Chunliu Dong

**Affiliations:** a College of Veterinary Medicine, Northeast Agricultural University; Harbin, Harbin, China; b Heilongjiang Key Laboratory for Animal Disease Control and Pharmaceutical Development, Northeast Agricultural University, Harbin, P.R. China

**Keywords:** Antibiotic adjuvant, *Escherichia coli*, bacterial resistance, florfenicol, arginine

## Abstract

The persistent escalation of antibiotic resistance poses a severe threat to global public health. As a common opportunistic pathogen, the rapid development of resistance in *Escherichia coli* (*E. coli*) poses severe challenges to clinical anti-infective therapy. Against this backdrop, the combined use of amino acids and antibiotics is considered an effective strategy against multidrug-resistant bacterial infections. This study investigated the antibacterial efficacy and mechanism of action of the combination of arginine (Arg) and florfenicol (FFC) against resistant *E. coli*. Results indicate that Arg significantly enhances the bactericidal activity of FFC against resistant *E. coli* both *in vivo* and *in vitro*, successfully reversing the resistance phenotype. Mechanistic studies reveal that Arg disrupts fatty acid β-oxidation by inhibiting the arginine succinyl-transferase (AST) pathway, leading to excessive free fatty acid (FFA) accumulation. This triggers membrane structural damage, energy metabolism disorders, and oxidative stress. Concurrently, Arg inhibits the expression and function of multidrug efflux pumps, such as AcrAB-TolC and EmrAB-TolC, reducing FFC efflux and increasing its intracellular concentration. This study systematically reveals a novel pathway whereby Arg synergistically enhances the antibacterial activity of FFC through dual mechanisms of metabolic reprogramming and efflux pump inhibition, providing theoretical support for the development and application of amino acid compounds as antibiotic adjuvants.

## Introduction

Antibiotics are critical tools for treating bacterial infections [1]. However, the misuse of antibiotics in human health care and livestock disease control has become increasingly severe, leading to the gradual development of antimicrobial resistance (AMR) in bacteria under prolonged selective pressure [2,3]. According to the *Review on Antimicrobial Resistance* (commonly known as the O’Neill Report), if current misuse trends persist, AMR could cause 10 million deaths annually by 2050 [4,5]. *Escherichia coli* (*E*. *coli*) is a clinically common Gram-negative rod-shaped bacterium. Under normal conditions, *E. coli* serves as a commensal colonizer in human and animal intestines without pathogenicity [6]. However, when host immunity is compromised or gastrointestinal barriers are disrupted, it can become an opportunistic pathogen, causing localized or systemic infections in the urinary tract, respiratory tract, peritoneum, and other sites [7]. In animal husbandry, pathogenic *E. coli* is one of the primary pathogens causing enteritis, urinary tract infections, septicemia, peritonitis, meningitis, and other diseases in livestock and poultry, resulting in significant economic losses to the industry [8]. Florfenicol (FFC) is a commonly used antibiotic in veterinary clinical practice for treating *E. coli* infections [9]. In China alone, its usage reached 2134 t in 2019 [10]. However, escalating resistance issues have diminished the original antibacterial efficacy of FFC. For instance, recent antimicrobial susceptibility testing on 220 pig-derived *E. coli* strains in China by Lei Zhen et al. revealed that 82.7% were resistant to FFC, with 41.3% showing high-level resistance [11]. Research by Li et al. indicates that the long-term misuse of FFC in domestic livestock farming has led to an explosive spread of resistant strains. The resistance rate among isolated pig-derived *E. coli* strains has exceeded 90% [12]. Currently, antibiotic resistance has become one of the major threats to global public health and development, while new drug development faces practical challenges, such as high costs, technical complexity, and lengthy timelines [13]. Therefore, developing innovative therapeutic strategies that enhance the efficacy of existing antibiotics has become an urgent necessity to address the resistance crisis.

Compared to developing new antibiotics, adding antimicrobial adjuvants offers a novel approach to repurpose existing drugs that balances efficiency, cost, and time [14,15]. Recent studies increasingly reveal a close connection between bacterial resistance and metabolic pathways. As core nodes in metabolic networks and key regulatory factors, amino acids have become crucial components of antimicrobial adjuvants [15]. l-Lysine enhances the bactericidal activity of aminoglycosides by interfering with the proton motive force, thereby promoting aminoglycoside uptake in *Acinetobacter baumannii* (*A*. *baumannii*) [16]; glutathione enhances the bactericidal effect of meropenem against *Klebsiella* pneumoniae (*K*. *pneumoniae*) by interfering with glycerophospholipid metabolism and increasing cell membrane permeability [17]; D-amino acids enhance the susceptibility of pathogenic *E. coli* to tetracycline and amikacin by inhibiting biofilm formation [18]. Currently, exogenous amino acid supplementation combined with antimicrobial drugs has become one of the primary approaches to address bacterial resistance.

Extensive research reveals that arginine (Arg), as a key-signaling molecule in bacterial metabolic networks, can directly or indirectly influence resistance phenotypes by reshaping intracellular metabolic pathways [19–22]. Han et al. [23] discovered that in *A. baumannii*, Arg metabolism can influence the dependence of the bacterium on polymyxin by regulating fatty acid metabolism pathways. Specifically, when the first key gene *astA* in the Arg succinyl-CoA synthetase pathway is deleted, the expression of genes involved in fatty acid β-oxidation is significantly downregulated [23]. Inhibition of the fatty acid β-oxidation pathway leads to substantial accumulation of free fatty acids (FFAs) within bacterial cells. The fatty acid degradation pathway is a core pathway for maintaining bacterial physiological functions, playing an irreplaceable role in cell membrane homeostasis and signal transduction. When this pathway is disrupted, triggering abnormal intracellular FFA accumulation, excess FFAs disrupt membrane integrity and lipid composition. This leads to membrane structural damage and ion leakage, exacerbating oxidative stress responses [24–26]. In *E. coli*, excess FFAs not only directly damage membrane structures, but also activate bacterial stress response pathways [24]. Medium-chain fatty acids such as decanoic acid and their derivatives can disrupt *E. coli* membrane integrity, causing ion leakage and structural damage [27]. Furthermore, arachidonic acid has been demonstrated to enhance the antimicrobial efficacy of aminoglycoside antibiotics by disrupting the cell membrane of *Staphylococcus aureus* (*S. aureus*), thereby promoting passive transmembrane transport [28]. Based on these findings, Arg holds promise as an antibiotic adjuvant for synergistically enhancing antimicrobial therapeutic outcomes. In conclusion, through both *in-vitro* and *in-vivo* experiments, this study systematically investigated the antibacterial effect of arginine combined with FFC on *E. coli*, and further clarified its mechanism of action by combining proteomics analysis. This provides new ideas for the prevention and treatment of infections caused by drug-resistant *E. coli*.

## Materials and methods

### Strains and growth conditions

The *E. coli* strain ATCC 25,922 used in this study was purchased from the American Type Culture Collection. Other strains were isolated from clinical samples by the Department of Veterinary Pharmacy at Northeast Agricultural University, including *Streptococcus* suis (*S*. *suis*), *S*. aureus, *K*. pneumoniae, and *E. coli*. Detailed strain information is provided in Table S1. Antimicrobial susceptibility testing was conducted in Mueller–Hinton (MH) broth. Unless otherwise specified, the growth conditions of strains were as follows: *S. suis* in Todd Hewitt (THB) broth, *S. aureus* on Tryptic Soy Agar (TSA), and *K. pneumoniae* and *E. coli* in Luria–Bertani (LB) medium, all incubated at 37°C with shaking.

### Antibiotics and chemicals

FFC (73231-34-2) was purchased from Shanghai Aladdin Biochemical Technology Co. Ltd., and L-Arg (74-79-3) was purchased from Dalian Meilun Biotechnology Co. Ltd.

### Minimum inhibitory concentration (MIC) determinations

Following CLSI standards (CLSI, 2024), the MIC of FFC against strains was determined using the broth microdilution method in MH medium. After incubation at 37°C in a constant-temperature incubator for 16–20 h, the MIC value was determined as the lowest drug concentration without visible turbidity. During the experiment, *E. coli* ATCC 25922 was concurrently used as a quality control strain to ensure its MIC value remained within the range of 2–8 μg/mL specified by CLSI, thereby validating the accuracy of the results. All experiments were repeated three times, and the final results were recorded as the average value.

### Checkerboard assay

Following CLSI standards (CLSI, 2024), the combined antimicrobial effect was determined using the checkerboard microdilution method. First, strains were cultured overnight to the logarithmic phase, adjusted to a turbidity of 0.5 McFarland units, and then diluted 1:300 in MH broth to 5 × 10^5^ CFU/mL. In a 96-well plate, establish a horizontal gradient of FFC concentrations and a vertical gradient of Arg concentrations. Add 100 μL of the drug combination to each well, followed by 100 μL of bacterial suspension (final bacterial concentration 5 × 10^5^ CFU/mL). Concurrently establish positive controls (bacteria-containing, drug-free), negative controls (drug-free, bacteria-free), and solvent controls (DMSO final concentration ≤1%). After incubation at 37°C for 18–20 h, growth inhibition was determined by visual assessment of no turbidity and OD_600_ < 0.1. The FICI index was calculated using the following formula: FICI = (MICA combination/MICA alone) + (MICB combination/MIB alone). FICI ≤ 0.5, 0.5–1, 1–4, and >4 were defined as synergistic, additive, no interaction, and antagonistic effects, respectively.

### Survival assay

Dilute the bacterial culture incubated for 16 h at a ratio of 1:1000 into the corresponding medium. Incubated until the OD_600_ reaches 0.5, then collect the culture at the exponential phase. Four control groups were established: blank control, Arg monotherapy, FFC monotherapy, and combination therapy. The collected cultures were resuspended in the respective media, drugs were added according to group assignments, and incubated at 37°C for 24 h. The bacterial suspensions were diluted with PBS and spread onto corresponding agar plates. After 24 h of incubation, colony counts were performed. Bacterial survival rate was calculated as the ratio of viable bacteria in the treated group to the blank group. The experiment was repeated three times, and results are expressed as mean ± standard deviation (SD).

### Time–kill assay

Using an initial bacterial concentration of 1.5 × 10^6^ CFU/mL, the experiment was divided into control, Arg monotherapy, FFC monotherapy, and combination therapy groups. Treatments included 4 μg/mL FFC, various concentrations of Arg, and combinations thereof. Samples were collected at 0, 2, 4, 8, 12, and 24 h. Viable cell counts were determined via dilute-plate counting. Bacterial density is presented relative to the starting inoculum at 0 h rather than to a growing control. A time–kill curve was plotted with time on the *x*-axis and viable cell count on the *y*-axis [29].

### Animal ethics approval

Animal experiments in this study were approved by the Animal Ethics Committee of Northeast Agricultural University (Number: NEAUEC20240397). All procedures adhered to ethical standards for laboratory animal welfare, minimizing animal harm to the greatest extent possible. This study adhered to the “ARRIVE” guidelines.

### Galleria mellonella (G. mellonella) larvae infection model

Sixty *G. mellonella* larvae (purchased from Tianjin Huiyude Biotechnology Co. Ltd.) were randomly divided into 6 groups of 10 larvae each. Except for the control group, the remaining groups were modeled by injecting 10 μL of 1 × 10^7^ CFU/mL *E. coli* FS95 into the right hind leg. Two hours post-infection, the treatment groups received injections of either Arg (15 mg/kg), FFC (5 mg/kg), or a combination of both (15 mg/kg +5 mg/kg) into the right hind leg. Larvae were cultured at 37°C with 5% CO_2_ for 120 h. Survival status was recorded every 24 h (no response to touch indicated death), and the 5-d survival rate was calculated.

### Murine bacterial peritonitis model

Thirty-five female ICR mice (18–20 g) from Liaoning Changsheng Biotechnology Co. Ltd. were randomly assigned to five groups of seven mice each. Cages were clearly labeled to avoid confusion. Group allocation and experimental procedures were performed in a blinded manner, with only the data analyst aware of the group assignments. The control group received an intraperitoneal injection of 100 µL saline, while the other groups were injected with 100 µL of a 1 × 10^9^-CFU/mL *E. coli* FS95 suspension to establish the model. Two hours post-infection, model groups received equal volumes of saline, while treatment groups received intraperitoneal injections of Arg (15 mg/kg), FFC (20 mg/kg), or combined therapy (15 mg/kg + 20 mg/kg). After 48 h, mice were deeply anesthetized by intraperitoneal injection of pentobarbital sodium (50 mg/kg) and then euthanized by cervical dislocation following AVMA guidelines. Heart, liver, spleen, lung, and kidney tissues were immediately collected. A portion was homogenized in physiological saline, appropriately diluted, and subjected to plate count methods to determine CFU counts in each tissue. Another portion of organs was preserved in 4% paraformaldehyde followed by hematoxylin and eosin (H&E) staining for pathological analysis.

### Whole-genome sequencing and genetic background analysis of bacterial strains

Third-generation single-molecule long-read sequencing of fluoroquinolone-resistant *E. coli* FS95 was performed using the PacBio Sequel II platform. Illumina NovaSeq second-generation sequencing data were integrated for error correction. The complete genomic sequences of the chromosome and plasmid (final assembly) were assembled using Canu v2.2 software. Gene annotation was performed using Prokka v1.14.6. Antibiotic resistance genes were screened using ResFinder v4.1 and CARD v3.2.9 databases. Phylogenetic relationships and genetic characteristics of the strain were analyzed through BLAST alignment.

### Proteomics analysis (DIA quantification)

*E. coli* FS95 was cultured in shaking conditions for 3 h until the exponential growth phase. Subsequently, either FFC (4 μg/mL) or Arg + FFC (40 mM + 4 μg/mL) was added, and the culture was incubated at 37°C with shaking for 6 h. The cells were harvested by centrifugation at 5500 rpm for 5 min and washed twice with PBS. The cell pellets were transported to the laboratory, rapidly frozen in liquid nitrogen, and stored for later use. Bacterial proteins were lysed, reduced, alkylated, and trypsin-digested. DIA data acquisition was performed using an EASY-nLC 1200 nanoliter liquid chromatography system coupled with a Q Exactive HF-X mass spectrometer. Raw data were analyzed for database searching and quantification using DIA-NN software (v1.8) and the UniProt *E. coli* database.

### Targeted FFA metabolism analysis

Activated *E. coli* FS95 cultures were grown in 50 mL medium for 3 h. One group received FFC (4 μg/mL) treatment, while another received combined Arg + FFC (40 mM + 4 μg/mL) treatment. After 6 h of incubation at 37°C, cells were harvested by centrifugation and washed twice with PBS. Following centrifugation, targeted metabolomics analysis of FFAs was performed. Samples underwent MTBE-methanol extraction, alkaline saponification, and methyl esterification before analysis via GC–MS (Agilent 8890-7000E, DB-5 MS column). Identification was conducted using the NIST database, with quantification via internal standard method. Differential fatty acids were identified using a threshold of |Log_2_FC| ≥1.

### Strain cultivation and treatment

*E. coli* FS95 strain was cultured for 3 h in four tubes containing 5 mL LB medium. Three tubes were supplemented with FFC (4 μg/mL), Arg (40 mM), or Arg + FFC (40 mM + 4 μg/mL) combinations, respectively, while the remaining tube served as a blank control. After incubation at 37°C for 6 h, cells were harvested by centrifugation at 5500 rpm for 5 min and washed twice with PBS.

### Gene expression was analyzed by RT-qPCR

Total RNA was extracted from the cultured bacterial suspensions using the M5 EASYspin Plus Kit (Mei5bio, Beijing). One microgram of RNA was reverse transcribed into cDNA using the cDNA Synthesis Kit (Mei5bio), followed by quantitative real-time PCR detection with SYBR Green Master Mix (Roche, Basel, Switzerland). Gene expression levels were normalized using 16S rRNA as an internal control. Specific primer sequences are detailed in Table S2 (synthesized by Genesoul Technology Co., Ltd., Harbin). Relative gene expression was calculated using the 2^(−ΔΔCt) method.

### Enzyme activity assay and lipid peroxidation quantification

Bacterial cultures were harvested and astA, fadA, fadB, fadE, superoxide dismutase (SOD), and catalase (CAT) activities were measured according to the Mlbio kit (Shanghai) protocol. SOD activity was measured by inhibiting the NBT photoreduction reaction (1 enzyme activity unit = 50% inhibition rate). CAT activity was calculated based on H_2_O_2_ decomposition rate at 240 nm. Lipid peroxidation levels were determined using the thiobarbituric acid (TBA) assay. All assays included three biological replicates; data are presented as mean ± standard deviation.

### Transmission electron microscopy analysis

The cultured bacterial suspension was fixed overnight at 4°C with 2.5% glutaraldehyde. Overnight samples underwent sequential dehydration with 30% to 100% ethanol solutions for 15 min each. Subsequent dehydration was performed twice with 100% acetone. Dehydrated specimens were permeated and embedded in a mixture of acetone and Epon 812 (1:1, 1:3, v/v, 30 min). Embedded blocks were trimmed, sectioned, and double-stained with uranium acetate and lead citrate. Finally, morphological and structural changes in bacterial cells were observed via transmission electron microscopy.

### Bacterial viability analysis

In *E. coli* viability assays, DAPI acts as an AT-sequence-specific minor groove binder to DNA, permeating bacterial cells to stain nucleic acids regardless of membrane integrity. PI functions as a parallel-intercalating agent for DNA double helices, penetrating only damaged cell membranes to stain nucleic acids. By analyzing the fluorescence images of both dyes, the ratio of membrane-damaged bacteria to the total bacterial population can be calculated as an indicator of membrane damage. Add 7.5 μL PI to 1 mL of the cultured bacterial suspension, incubate for 10 min, centrifuge at 6000 rpm for 5 min, discard the supernatant, add 1 mL PBS and re-centrifuge, then add 200 μL DAPI and incubate for 15 min before observing under a microscope.

### Detection of cellular inner and outer membrane permeability

Pick a single colony of *E. coli* FS95 and culture it overnight in LB medium. The next day, collect the bacterial culture, wash it three times with 5 mM HEPES and 5 mM glucose buffer (pH 7.2), and adjust it to an OD_600_ of 0.5. For outer membrane permeability testing, incubate a portion of the culture with 10 μM NPN in the dark for 15 min. Subsequently, add either FFC (4 μg/mL), Arg (40 mM), or Arg + FFC (40 mM +4 μg/mL). Immediately, monitor fluorescence changes for 60 min using an enzyme-linked immunosorbent assay reader at an excitation wavelength of 350 nm and an emission wavelength of 420 nm. Separately, a portion of the bacterial culture was incubated in the dark with 7.5 μg/mL PI for 15 min to assess inner membrane permeability. After adding the same drug combinations at equivalent concentrations, fluorescence changes were monitored at an excitation wavelength of 535 nm and an emission wavelength of 615 nm. For the outer membrane permeability assay, EDTA (5 mM) was used as a positive control. For the inner membrane permeability assay, Triton X-100 (0.1%) was used as a positive control. All experiments included three biological replicates.

### Cell membrane fluidity assay

Take *E. coli* FS95 culture with OD_600_ = 0.5, add 10 μM Laurdan, incubate at 37°C in the dark for 10 min, wash three times with PBS, and divide into groups. Add FFC (4 μg/mL), Arg (40 mM), Arg + FFC (40 mM + 4 μg/mL), and benzyl alcohol (positive control). Incubate at 37°C for 35 min. Measure fluorescence at 460 nm and 500 nm excitation at 350 nm using a microplate reader. Repeat three times. Membrane fluidity was calculated using the Laurdan GP formula (GP = (*I*_460_ − *I*_500_)/(*I*_460_ + *I*_500_)).

### Cell membrane potential detection

Take *E. coli* FS95 culture with OD_600_ = 0.5, add 0.5 μM DiSC_3_(_5_) under dark conditions, and incubate at 37°C for 15 min. Subsequently, add FFC (4 μg/mL), Arg (40 mM), or Arg + FFC (40 mM + 4 μg/mL). Measure fluorescence changes in each group over 60 min using a microplate reader at an excitation wavelength of 622 nm and an emission wavelength of 670 nm [30]. CCCP (10 μM) was used as a positive control for membrane depolarization. All experiments were repeated three times.

### Proton drive detection

Take *E. coli* FS95 culture with OD_600_ = 0.*5*, mix thoroughly with 10 μM BCECF-AM, and incubate at 37°C in the dark for 15 min. Subsequently, add FFC (4 μg/mL), Arg (40 mM), or Arg + FFC (40 mM + 4 μg/mL). Measure fluorescence changes in each group over 60 min using a microplate reader at an excitation wavelength of 488 nm and an emission wavelength of 535 nm [3]. All experiments were repeated three times.

### Intracellular and extracellular ATP level detection

Take *E. coli* FS95 culture with OD_600_ = 0.5, add FFC (4 μg/mL), Arg (40 mM), or Arg + FFC (40 mM + 4 μg/mL), and continue incubation for 6 h. Detect extracellular and intracellular ATP levels in *E. coli* FS95 using an enhanced ATP detection kit (Beyotime).

### ROS level detection

Take *E. coli* FS95 culture with OD_600_ = 0.5, mix thoroughly with 10 μM DCFH-DA probe protected from light, and incubate by shaking at 37°C for 20 min. Resuspend in fresh PBS, then add FFC (4 μg/mL), Arg (40 mM), Arg + FFC (40 mM + 4 μg/mL), and the positive control Rosup reagent. Incubate in the dark at 37°C for 6 h. After centrifugation and washing, measure the fluorescence values of each group using a microplate reader at an excitation wavelength of 488 nm and an emission wavelength of 525 nm. All experiments were repeated three times.

### Intracellular FFC accumulation level detection

Take *E. coli* FS95 culture with OD_600_ = 0.*5*, add FFC (4 μg/mL), Arg (40 mM), or Arg + FFC (40 mM + 4 μg/mL), and incubate with shaking at 37°C for 30 min. Wash three times with PBS, resuspend in sterile ddH_2_O, sonicate cells, and centrifuge to collect supernatant. Finally, strictly follow the instructions of the FFC residue detection kit (Beyotime) to detect FFC accumulation in bacteria. Each experiment was repeated three times.

### Efflux pump function assay

Take OD _600_ = 0.5 *E. coli* FS95 culture was mixed with 2 μg/mL EtBr and incubated at 37°C for 1 h. After resuspension in fresh PBS, FFC (4 μg/mL), Arg (40 mM), Arg + FFC (40 mM + 4 μg/mL), and positive control CCCP (10 μg/mL) were added. Finally, monitor EtBr efflux over 1 h at excitation wavelength 530 nm and emission wavelength 600 nm using a microplate reader. All experiments were repeated three times.

### Statistical analysis

Statistical analysis was performed using SPSS version 26.0 software, and Student’s *t*-test was employed. Results are expressed as means ± standard deviations. Growth patterns were modeled using GraphPad Prism 10.0. The thresholds for statistical significance were defined as follows: ns for *p* > 0.05, * for *p* < 0.05, ** for *p* < 0.01, and *** for *p* < 0.001.

## Results

### In-vitro antibacterial study of Arg combined with FFC against *E. coli*

**Figure 1.**
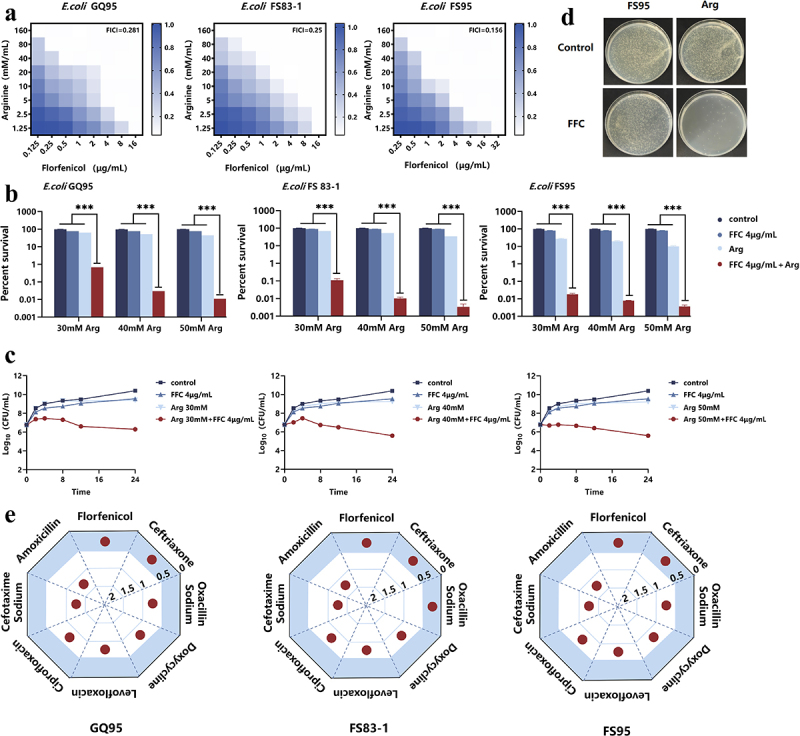
In vitro enhancement of the antibacterial activity of FFC against *E. coli* by Arg. (a) Synergistic effects of Arg and FFC on various clinically isolated *E. coli* strains (synergy defined as fractional inhibitory concentration index (FICI) ≤0.5, additive effect: 0.5 < FICI ≤ 1, no difference: 1 < FICI ≥ 4, antagonism: FICI > 4); (b) effect of 4 μg/mL FFC combined with 30, 40, or 50 mM Arg on survival rates of various clinically isolated *E. coli* strains; (c) time-dependent killing curves of *E. coli* FS95 exposed to 4 μg/mL FFC combined with 30, 40, or 50 mM arg; (d) verification of the antibacterial activity of the 4 μg/mL FFC and 40 mM arg combination against *E. coli* FS95 using the colony count method. (e) Synergistic and additive effects of Arg in combination with various antimicrobial agents against GQ95, FS83-1, and FS95. ns; *p* > 0.05; *p < 0.05; **p < 0.01; ***p < 0.001.

This study selected one standard *E. coli* strain (ATCC 25922) and three clinical isolates (FS 83-1, GQ95, FS95) to determine the minimum inhibitory concentration (MIC) of FFC against each strain. Results showed that only the standard strain was susceptible to FFC, while all three clinical isolates exhibited resistance (Table 1). To evaluate whether Arg and FFC exhibit synergistic antibacterial effects, the fractional inhibitory combination index (FICI) was determined using the checkerboard microdilution method. The results showed that the FICI values for the combination of Arg and FFC against GQ95, FS 83-1, and FS95 were 0.28, 0.25, and 0.15, respectively (Figure 1(a)), all meeting the criteria for synergistic activity (FICI ≤ 0.5). Further validation of the combination’s antibacterial activity via colony counting revealed that 30–50 mM Arg combined with 4 μg/mL FFC exhibited significant bactericidal effects against all resistant strains (Figure 1(b)). Based on these findings, FS95 (a strain with a well-defined genetic background) was selected as the subsequent test strain to determine the optimal in vitro combination concentrations. Analysis of time–kill curves (Figure 1(c)) revealed that 4 μg/mL FFC combined with 30, 40, or 50 mM Arg inhibited *E. coli* FS95 growth, with 40 mM Arg yielding the optimal effect (Figure 1(d)). This combination reduced colony counts by approximately 4 Log_10_, demonstrating potent synergistic bactericidal activity. Given the broad-spectrum antibacterial properties of FFC, this study further validated the synergistic effects of Arg combined with FFC against other clinically common pathogens (Table S3, Figures S1 and S2). Results demonstrated synergistic antibacterial activity against *S. suis*, *S. aureus*, and *K. pneumoniae*, suggesting its potential broad-spectrum application value. Additionally, we determined whether Arg could enhance the in vitro antibacterial activity of other antimicrobial agents. Results demonstrated that Arg not only enhanced the antibacterial activity of FFC but also exhibited significant synergistic effects with ceftriaxone. When combined with oxacillin sodium, doxycycline, tetracycline, levofloxacin, or ciprofloxacin, Arg produced additive effects (Figure 1(e), Tables S4 and S5). These findings suggest that Arg may serve as a broad-spectrum antimicrobial synergist, though its mechanism of action requires further investigation.

**Table 1.** MIC values of *E. coli* for FFC in this study.

Strains	MICs of FFC on strains (μg/ml)	Interpretation
*E. coli* ATCC25922	4	S
*E. coli* FS83-1	16	R
*E. coli* GQ95 *E. coli* FS95	32 32	R R

MIC inflection point of FFC: S ≤ 4; I = 8; *R* ≥ 16. S (Sensitive) indicates the drug effectively inhibits microorganisms at the recommended dose; I (intermediate) indicates the drug requires a higher-than-standard dose or specific conditions to be effective; R (resistant) indicates the drug fails to exert antimicrobial activity even at high concentrations due to the presence of resistance mechanisms.

### In-vivo antibacterial testing of the combination of Arg and FFC against *E. coli*

**Figure 2.**
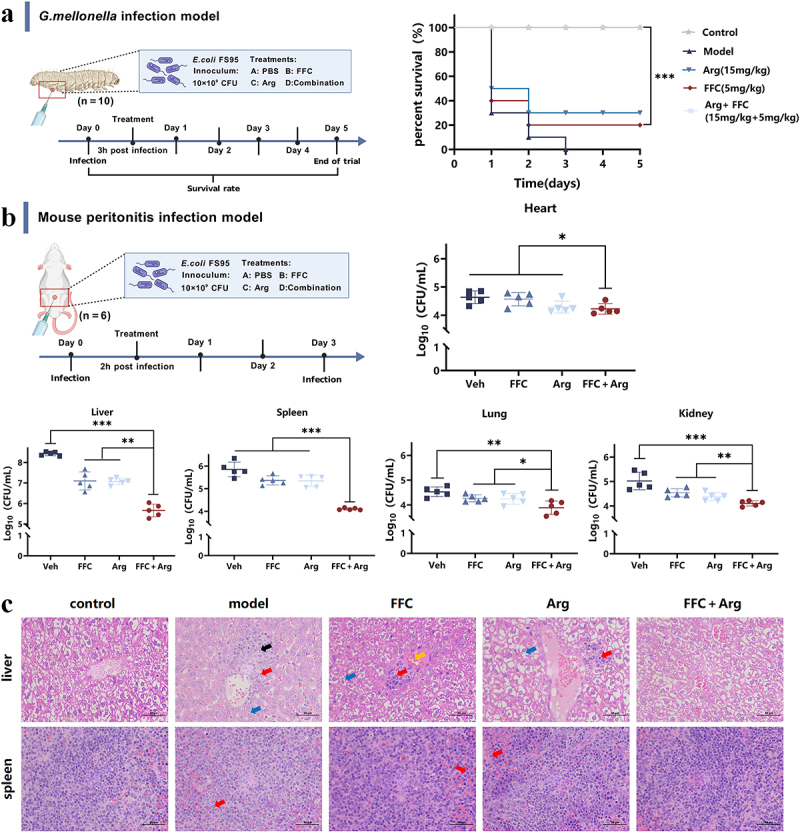
In vivo enhancement of the antibacterial activity of FFC against *E. coli* by Arg. (a) Experimental protocol for the *G. mellonella* infection model and survival curve analysis of infected larvae under different treatments (*n* = 10). (b) Bacterial peritonitis infection model in mice and bacterial load in infected mouse heart, liver, spleen, lung, and kidney tissues under different treatments (*n* = 5); (c) H&E staining results of liver and spleen in the bacterial peritonitis infection model. Liver: connective tissue proliferation (black arrow), vascular congestion (orange arrow), granulocyte infiltration (red arrow), hepatocyte edema (blue arrow); spleen: granulocyte infiltration (red arrow). All data are presented as mean ± standard deviation. *p*-Values were determined using an unpaired two-tailed Student’s *t*-test. ns, *p* > 0.05; *p < 0.05; **p < 0.01; ***p < 0.001.

Given the significant *in-vitro* antibacterial activity of Arg combined with FFC against *E. coli*, a *G. mellonella* larva infection model and a mouse bacterial peritonitis model were established to evaluate its in vivo antibacterial efficacy. In the *G. mellonella* larval infection model (Figure 2(a)), survival rates after monotherapy with Arg or FFC were 30% and 20%, respectively; however, combined therapy achieved a 100% survival rate (Figure 2(a)). These results indicate that Arg significantly enhances the *in-vivo* antibacterial activity of FFC (*p* < 0.01). In a mouse bacterial peritonitis model (Figure 2(b)), compared to the model group and monotherapy groups, combined Arg and FFC treatment significantly reduced bacterial load in various mouse organ tissues (Figure 2(b)), with more pronounced decreases observed in liver and spleen (*p* < 0.01). Further pathological examination of liver and spleen via H&E staining (Figure 2(c), scale bar = 200 μm; Figure S3, scale bar = 50 μm) revealed significant improvements in the combined group compared to the model and monotherapy groups. These improvements included enhanced central vein structure, reduced vascular congestion, decreased inflammatory infiltration, improved connective tissue organization, and normalized hepatocyte morphology in the liver. In the spleen, the combined treatment group showed significant improvements in white pulp architecture, red–white pulp boundary, megakaryocyte count, sinus congestion, and granulocyte infiltration.

### Proteomics-based investigation of potential mechanisms underlying Arg-FFC synergy in *E. coli*

**Figure 3.**
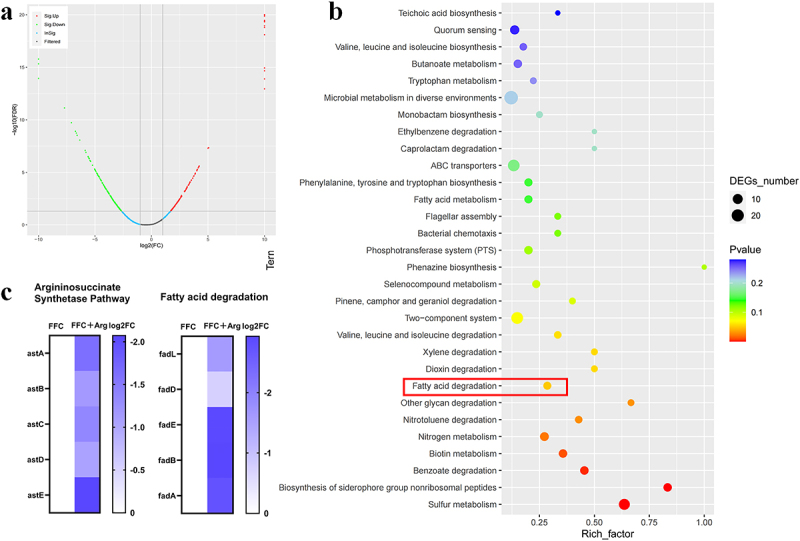
Proteomics-based differential expression and pathway analysis of Arg-FFC synergistic effects. (a) Volcano plot of differentially expressed proteins (DEPs) between Arg-FFC combined treatment and FFC alone; (b) KEGG pathway enrichment diagram of DEPs in the Arg-FFC combined treatment group versus FFC alone; (c) heatmap of differential protein expression related to the fatty acid β-oxidation pathway and Arg-succinate transferase-mediated metabolic pathway.

To clarify the genetic background of the laboratory-derived clinical *E. coli* isolate (designated FS95) used in subsequent experiments, whole-genome sequencing was performed. The raw whole-genome sequencing data have been submitted to the NCBI SRA database (project number PRJNA1236709, sample number SAMN47402138; second-generation and third-generation data numbers are SRR32725481 and SRR32725480). Building upon this, to investigate the potential mechanisms underlying the observed synergistic effects, this study used *E. coli* FS95 as the research subject. Separate treatment groups were established: an FFC-only group and an Arg-FFC combined treatment group. After 6 h of drug exposure, the proteomic profiles of bacteria in both groups were analyzed. Compared to FFC monotherapy, combined treatment significantly regulated the bacterial proteome expression profile, with 130 proteins upregulated and 230 downregulated (Figure 3(a)). KEGG pathway enrichment analysis of differentially expressed proteins (DEPs) revealed these DEPs primarily participated in key biological pathways, such as bacterial fatty acid degradation and amino acid metabolism. Further analysis revealed that combined treatment significantly suppressed the fatty acid β-oxidation pathway and the metabolic pathway mediated by Arg succinate transferase (Figure 3(b,c)).

### Arg-FFC inhibits fatty acid β-oxidation in *E. coli* by suppressing the argininosuccinate synthase pathway

**Figure 4.**
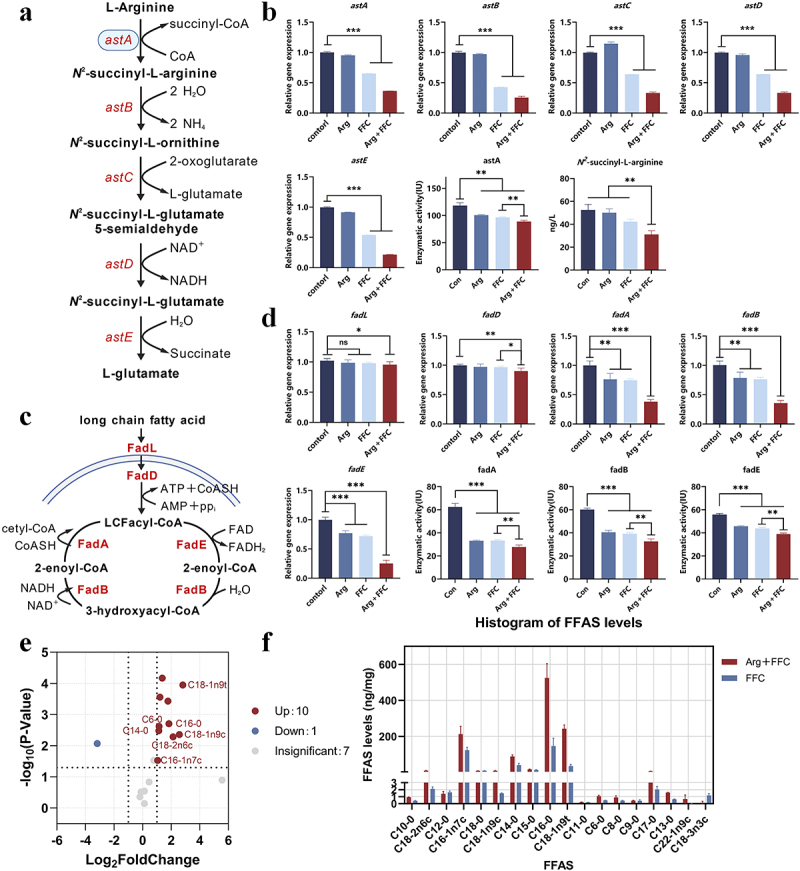
Synergistic inhibition of Arg metabolism and fatty acid oxidation in *E. coli* FS95 by combined treatment. (a) Schematic diagram of the Arg succinyl-CoA synthetase pathway; (b) changes in transcription, enzyme activity, and metabolite levels of the Arg succinyl-CoA synthetase pathway; (c) schematic diagram of the fatty acid β-oxidation pathway; (d) changes in transcription and enzyme activity levels of the fatty acid β-oxidation pathway; (e) volcano plot of differential metabolites of FFAs; (f) bar chart of FFA levels. ns, *p* > 0.05; *p < 0.05; **p < 0.01; ***p < 0.001.

Based on proteomics analysis, this study hypothesizes that the combined treatment (Arg-FFC) may exert synergistic effects by inhibiting the Arg-succinate transferase-mediated metabolic pathway and the fatty acid β-oxidation pathway in *E. coli* FS95. To validate this hypothesis, we first experimentally verified the Arg-succinate synthase pathway. Results showed that compared to FFC treatment alone, the transcription levels of key genes in this pathway (*astA*, *astB*, *astC*, *astD*, *astE*) were significantly downregulated in the combined treatment group. Simultaneously, the activity of the Arg succinate lyase encoded by *astA* was markedly reduced, and the accumulation of the intermediate metabolite *N*-succinyl-L-Arg was significantly decreased (Figure 4(a,b)), indicating that this pathway was indeed inhibited.

Previous studies have reported that knocking out the *astA* gene in Gram-negative bacilli can inhibit fatty acid β-oxidation [27], suggesting that this regulatory mechanism may be conserved in Gram-negative bacteria. Based on the clear inhibition of *astA* and related pathways observed in the combined treatment in this study, we further speculate that it may also indirectly affect fatty acid β-oxidation. Experimental results showed that transcription levels of multiple key fatty acid β-oxidation genes (*fadL*, *fadD*, *fadA*, *fadB*, *fadE*) were significantly reduced in the combined treatment group, and the activities of enzymes encoded by fadA, fadB, and fadE were also markedly decreased (Figure 4(c,d)). Targeted metabolomics analysis further revealed a significant overall increase in FFA levels in the combined treatment group (Figure 4(e,f)). Collectively, this study demonstrates at multiple levels that Arg-FFC cotreatment indirectly inhibits fatty acid β-oxidation by suppressing the Arg succinyl-CoA synthetase pathway.

### Mechanisms of membrane damage and oxidative stress mediated by FFA accumulation

**Figure 5.**
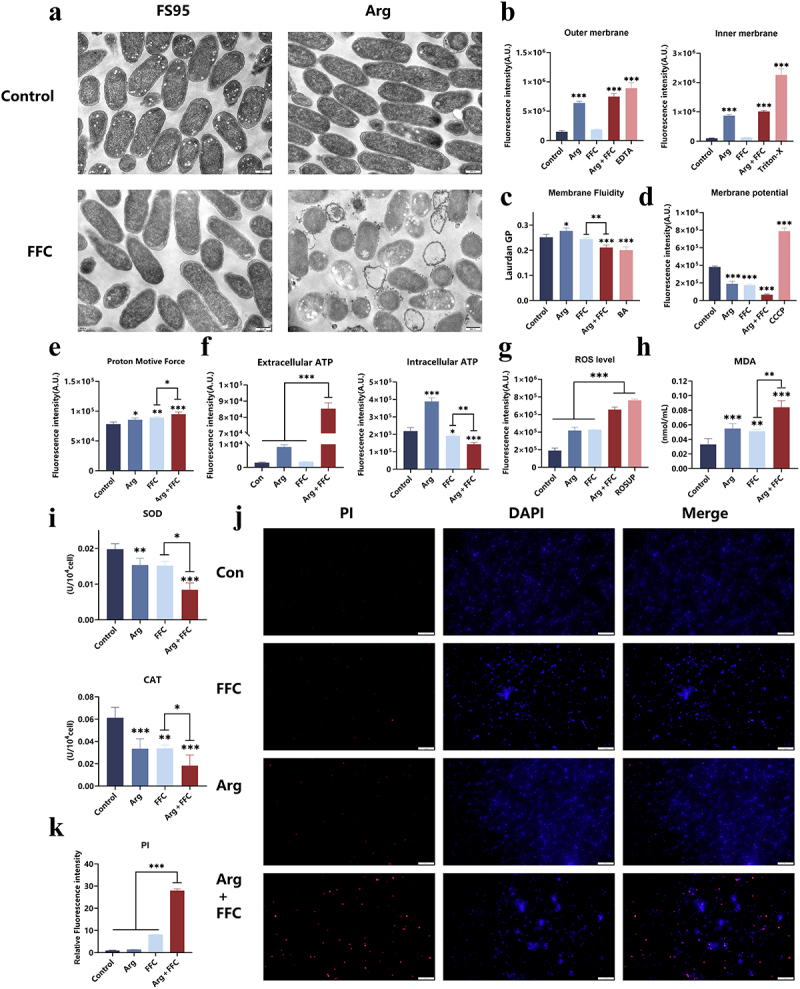
Excessive FFAs induce membrane damage and oxidative stress in *E. coli* FS95. (a) Transmission electron microscopy revealing ultrastructural changes; (b) NPN and PI fluorescent probes for outer and inner membrane permeability; (c) Laurdan fluorescence assay for membrane fluidity; (d) DiSC3(5) fluorescent probe for membrane potential (Δ*ψ*); (e) BCECF-AM fluorescent probe for transmembrane proton gradient (Δ*pH*); (f) ATP assay kit for intracellular and extracellular ATP levels; (g) DCFH-DA assay for intracellular ROS levels; (h) thiobarbituric acid assay for malondialdehyde (MDA) content; (i) superoxide dismutase (SOD) and catalase (CAT) activity assays; (j) DAPI/PI dual-staining fluorescence imaging analysis of bacterial mortality. ns, *p* > 0.05; *p < 0.05; **p < 0.01; ***p < 0.001.

Targeted metabolomics revealed that combined treatment induced significant accumulation of multiple FFAs (e.g. palmitic acid, oleic acid, and linoleic acid) within bacterial cells (Figure 4(e,f)). Extensive studies indicate that such compounds can disrupt cell membrane structure and induce oxidative stress [31]. Based on this, we hypothesize that Arg-FFC exerts synergistic bactericidal effects by inducing FFAs accumulation. To investigate the effects of FFAs on *E. coli* cell membranes, we conducted relevant experiments. Transmission electron microscopy (TEM) revealed that Arg-FFC treatment caused significant rupture of the FS95 *E. coli* cell membrane, accompanied by leakage of intracellular material (Figure 5(a)). Membrane permeability assays further demonstrated that this treatment simultaneously disrupted the integrity of both the outer- and inner-bacterial membranes, while increasing membrane fluidity (Figure 5(b,c)). Such membrane structural damage inevitably disrupts physiological functions. Experiments revealed that the combination treatment led to a significant decrease in DiSC_3_(5) fluorescence, indicating membrane hyperpolarization (increased Δ*ψ*), while the BCECF–AM ratio was significantly increased, reflecting an elevated Δ*pH* component (Figure 5(d,e)). These results indicate that the combination treatment triggered hyperpolarization of Δ*ψ* and elevation of Δ*pH*, rather than simple proton motive force (PMF) dissipation. Notably, such alterations established a proton futile cycle that uncoupled PMF from ATP synthesis, ultimately resulting in energy collapse despite increased transmembrane gradients. Additionally, intracellular ATP levels plummeted, while extracellular ATP levels significantly increased (Figure 5(f)), indicating physical leakage of ATP due to membrane damage, consistent with the membrane structural disruption observed by TEM (Figure 5(a)). This depletion of the intracellular ATP pool signals collapse of the energy metabolism system. Moreover, FFA accumulation triggered intense oxidative stress. Intracellular reactive oxygen species (ROS) levels markedly increased (Figure 5(g)), leading to substantial accumulation of the lipid peroxidation product malondialdehyde (MDA) (Figure 5(h)), indicating substantial oxidative damage to the bacterial cell membrane. More critically, the activities of superoxide dismutase (SOD) and catalase (CAT) were both significantly inhibited (Figure 5(h,i)), indicating that the bacterial antioxidant system had been inactivated, rendering oxidative damage irreversible. Ultimately, fluorescence microscopy with DAPI (stains all bacteria, blue) and PI (stains membrane-damaged bacteria, red) revealed that the combined effects of membrane damage and oxidative stress led to massive bacterial death, as indicated by the increased proportion of PI-positive cells (Figure 5(j,k)). In summary, the experiments revealed that combined drug treatment induces intracellular FFA accumulation in *E. coli*, subsequently disrupting cell membrane structural integrity and function while triggering irreversible oxidative stress, ultimately leading to massive bacterial death. To clarify the pivotal role of FFA accumulation in these phenotypes, we performed reverse validation by exogenously adding palmitic acid, oleic acid, and linoleic acid, which are three fatty acids that significantly accumulate within the bacteria. Experimental results demonstrated that the individual use of these FFAs not only directly caused a significant reduction in bacterial survival rate (Figure S4a) but also reproduced the core phenotypes induced by Arg-FFC combined treatment, including impaired cell membrane structural integrity (Figure S4b) and significantly elevated intracellular reactive oxygen species (ROS) levels (Figure S4c). These findings provide strong evidence that the synergistic bactericidal effect of Arg-FFC is mediated by inducing intracellular FFA accumulation.

### Arg-FFC inhibits drug efflux pump function and increases intracellular FFC accumulation

**Figure 6.**
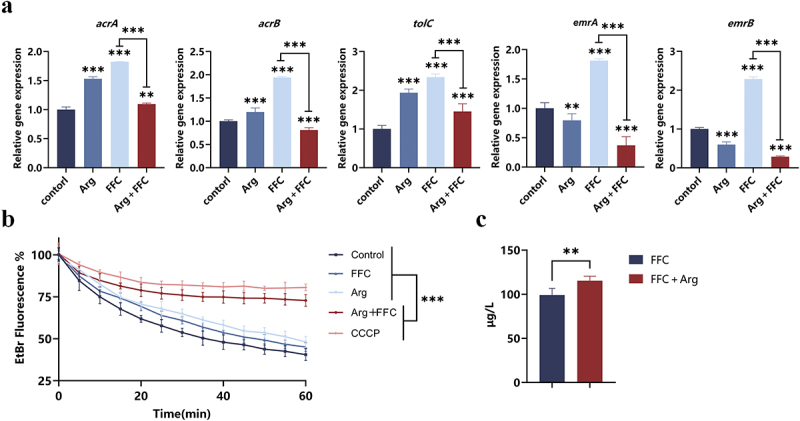
Arg enhances intracellular accumulation of FFC by inhibiting efflux pump function. (a) Relative transcription levels of efflux pump genes (*acrA*, *acrB*, *tolC*, *emrA*, *emrB*); (b) time-course changes in intracellular ethidium bromide (EtBr) fluorescence intensity across treatment groups; (c) intracellular FFC concentration measurement. ns, *p* > 0.05; *p < 0.05; **p < 0.01; ***p < 0.001.

Beyond interfering with metabolic pathways, we investigated whether Arg-FFC enhances the antibacterial activity of FFC by inhibiting efflux pump function. qRT-PCR results showed that cotreatment significantly downregulated the transcriptional levels of efflux pump genes (*acrA*, *acrB*, *tolC*, *emrA*, *emrB*) in *E. coli* FS95 (Figure 6(a)). EtBr efflux assays further demonstrated higher intracellular fluorescence intensity and slower decay in the cotreated group (Figure 6(b)), confirming impaired efflux pump function. Additionally, intracellular FFC concentration measurements revealed significantly increased drug accumulation in the co-treated group (Figure 6(c)). Proteomics analysis further supported these findings (Figure S5), revealing that Arg-FFC treatment significantly reduced the expression levels of multiple efflux pump-related proteins, consistent with qRT-PCR and functional assay results. Collectively, these results demonstrate that Arg inhibits efflux pump expression and function, reducing FFC efflux and increasing its intracellular concentration, thereby enhancing bactericidal activity. This represents a synergistic mechanism independent of metabolic disruption.

## Discussion

In recent years, the global incidence of infections caused by drug-resistant bacteria has continued to rise, and the problem of antibiotic resistance has become increasingly severe. This situation highlights the limitations of current infection prevention and control strategies [32]. Particularly against the backdrop of bottlenecks in the development of new antibiotics, reshaping the therapeutic potential of existing antimicrobial drugs has become an important direction for addressing the challenge of resistance. Recent studies indicate that combining various amino acids with antibiotics can effectively reverse bacterial resistance [33–35], offering a strategy to restore antimicrobial activity in traditional drugs. Building on this, our research demonstrates that Arg significantly enhances the in vitro and in vivo antibacterial effects of FFC against resistant *E. coli*, successfully reversing its resistance phenotype. Furthermore, our article delves into the underlying mechanism of action.

Han et al. [27] reported that inhibition of the arginine succinyl-transferase (AST) pathway further impedes fatty acid β-oxidation, and this metabolic alteration directly influences the dependence of *A. baumannii* on polymyxin. This aligns with our proteomics findings, where exogenous Arg supplementation combined with FFC treatment significantly suppressed both the argininosuccinate synthetase pathway and fatty acid β-oxidation pathway in FFC-resistant *E. coli*. This finding reveals a close association between Arg metabolism and fatty acid metabolism, suggesting that this regulatory link may be the key mechanism by which Arg enhances the antibacterial activity of FFC against resistant *E. coli*. In our study, the expression levels of key genes in the arginyl succinyl-CoA synthetase pathway [36,37], astA enzyme activity decreased, and accumulation of the intermediate metabolite *N*-succinyl-L-Arg diminished. Based on the research conclusion proposed by Han et al. [27], inhibition of the arginine succinyltransferase pathway can hinder fatty acid β-oxidation, we speculate that the combined treatment’s inhibition of this pathway may indirectly affect the fatty acid β-oxidation process. Consistent with this hypothesis, the combined treatment significantly reduced the transcriptional levels of key fatty acid β-oxidation genes [38] and the activity of enzymes encoded by *fadA, fadB*, and *fadE*. Furthermore, targeted metabolomics revealed substantial accumulation of FFAs (such as palmitic acid, oleic acid, and linoleic acid), corroborating the functional impairment of this pathway. Previous studies have thoroughly demonstrated that excessive accumulation of FFAs in bacteria disrupts cell membrane integrity, interferes with energy homeostasis, and induces severe oxidative stress, thereby exerting antibacterial effects [39–41]. Hexanoic, octanoic, and decanoic acids significantly inhibit *E. coli* growth under low pH conditions and cause alterations in membrane fluidity and integrity [42]; palmitic acid induces rapid membrane depolarization, impaired macromolecular synthesis, and intracellular leakage in *S. aureus* [43]; myristic acid, palmitic acid, oleic acid, and lauric acid also exhibit significant antibacterial activity against *Staphylococcus* species [44]. The decreased ATP levels and ROS accumulation observed in this study align closely with the aforementioned mechanisms. Furthermore, exogenous addition of these FFAs reproduces the core phenotypes of combined treatment, further confirming the pivotal role of FFA accumulation in the synergistic bactericidal mechanism of Arg-FFC. Notably, FFC itself can also interfere with lipid metabolism [45–47]. As a key decomposition process of lipid metabolism, the functional state of fatty acid β-oxidation is directly regulated by the overall lipid metabolism. The addition of arginine may enhance this effect by synergistically inhibiting the AST pathway and fatty acid β-oxidation with florfenicol.

Beyond indirectly enhancing the antibacterial effect of FFC by disrupting metabolic homeostasis, this study also revealed that Arg suppresses the expression of multiple efflux pump genes. Combined with previous observations of Arg exhibiting synergistic effects with various antibiotics in vitro, we hypothesize that inhibition of efflux pump function may represent a key mechanism underlying the broad-spectrum antimicrobial sensitization of Arg. Specifically, combined treatment significantly suppressed the expression of multiple multidrug efflux pump genes in *E. coli*. Functional assays further confirmed markedly reduced efflux activity of ethidium bromide, leading to substantially increased intracellular accumulation of FFC. Notably, the classic RND family efflux pump AcrAB-TolC efficiently expels FFC [48,49]. Moreover, EmrAB-TolC can be compensatorily expressed to maintain resistance when AcrAB-TolC function is impaired [50]. The simultaneous inhibition of both efflux pump categories by the combined treatment fundamentally undermines bacterial efflux capacity. We speculate that this inhibitory effect may be an indirect consequence of the “metabolic disruption” induced by the combined treatment. Although the combination treatment elevated both components of PMF (Δ*ψ* and Δ*pH*) [51–53], the FFA-mediated proton leakage created a futile cycle that uncoupled PMF from ATP synthesis. Since efflux pumps require a coupled PMF to function efficiently, this energetic uncoupling likely contributes to impaired efflux pump activity. Notably, a recent study confirmed the central role of efflux pumps in FFC resistance. Kerek et al. [54] found that FFC stress induces mutations in efflux pump regulatory genes, such as *acrR* and *emrR* in *E. coli*, thereby activating multidrug efflux pumps including AcrAB-TolC, EmrAB-TolC, and MdtABC-TolC. This leads to cross-resistance against fluoroquinolones and cephalosporins. This study suggests that Arg may also effectively block this resistance pathway by directly inhibiting the expression and function of the aforementioned efflux pumps or by interfering with the activation of their regulatory networks, thereby restoring susceptibility to FFC.

**Figure 7.**
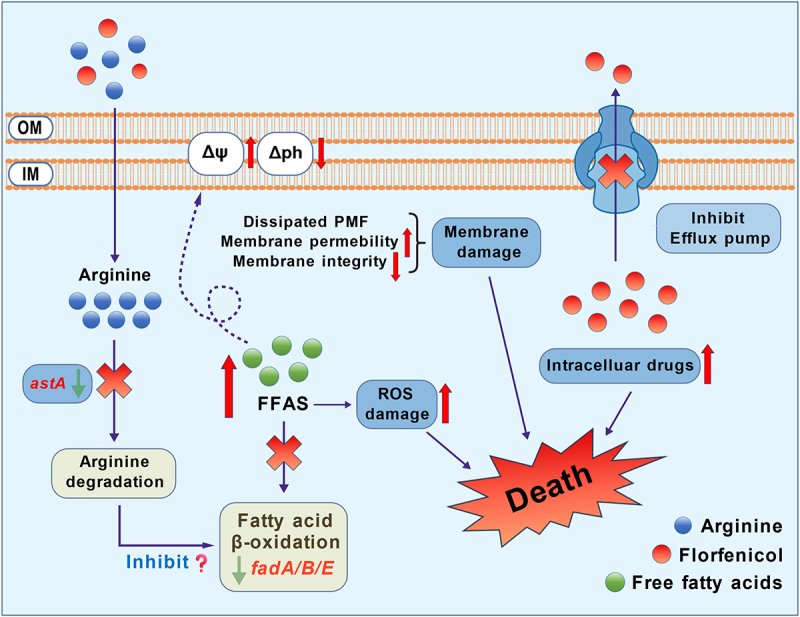
Schematic analysis of the synergistic mechanism between Arg and FFC.

In summary, our research elucidates a dual synergistic mechanism by which Arg restores FFC efficacy: (i) indirectly inhibiting fatty acid β-oxidation by suppressing the Arg succinyl-CoA transferase pathway. Inhibition of fatty acid oxidation leads to massive accumulation of FFAs, which cause bacterial membrane damage and oxidative stress, ultimately resulting in bacterial death. (ii) Arg inhibits efflux pump expression and function, reducing FFC efflux and increasing its intracellular concentration, thereby enhancing bactericidal activity (Figure 7). These findings not only provide a potential therapeutic strategy to overcome FFC resistance but also establish a theoretical foundation for further exploring amino acid-assisted antibiotic sensitization mechanisms.

## Conclusion

This study demonstrates that Arg is an effective antimicrobial potentiator, which significantly restores the antibacterial efficacy of FFC against resistant *E. coli* and provides a novel therapeutic strategy against drug-resistant bacterial infections. As a fundamental mechanistic investigation, it confirms that Arg enhances the bactericidal activity of FFC both *in vivo* and *in vitro*, and systematically elucidates its synergistic mechanism through dual pathways of “metabolic reprogramming” and “efflux pump inhibition.” However, this research remains limited in its direct applicability to clinical treatment of resistant infections. The optimal combination dosage of Arg and FFC, their pharmacokinetic interactions *in vivo*, and long-term safety profiles have yet to be determined. Therefore, further systematic studies – including in vivo pharmacodynamics, pharmacokinetics, and toxicology evaluations – are warranted to facilitate the translation of this combination strategy into clinical practice.

## Supplementary Material

Supplemental Material

## Data Availability

The data supporting this study are publicly available in the ScienceDB repository (https://doi.org/10.57760/sciencedb.34542) [55]. Whole-genome sequencing data of *E. coli* FS95 are deposited in NCBI SRA under BioProject PRJNA1236709. All other data are included in the main text or supplementary materials.
