# Supplementary material for: Synergistic antibacterial activity and mechanism of arginine combined with florfenicol against Escherichia coli
Source: Virulence. 2026 May 4;17(1):2666994. doi: 10.1080/21505594.2026.2666994 (PMC13154936; doi:10.1080/21505594.2026.2666994)
Supplement: Supplemental Material [file KVIR_A_2666994_SM7905.docx]

Table S1. All specific strain information used in this study

| strains | Source |
| --- | --- |
| *S. suis* 3-3-1 | Laboratory clinical isolation |
| *S. suis* 11 | Laboratory clinical isolation |
| *S. suis* 089 yellow 2-1 | Laboratory clinical isolation |
| *Staphylococcus aureus* ZYH 2-11 | Laboratory clinical isolation |
| *Staphylococcus aureus* ZYH 2-3 | Laboratory clinical isolation |
| *Staphylococcus aureus* ZYH 1-2 | Laboratory clinical isolation |
| *E. coli* ATCC 25922 | American Type Culture Collection |
| *E. coli* FS83-1 | Laboratory clinical isolation |
| *E. coli* GQ95 | Laboratory clinical isolation |
| *E. coli* FS95 | Laboratory clinical isolation |
| *K. pneumoniae* 090 blue | Laboratory clinical isolation |
| *K. pneumoniae* 1-20 C20 | Laboratory clinical isolation |
| *K. pneumoniae* 1-20 B23 | Laboratory clinical isolation |

Table S2. Primers used in real-time quantitative PCR

| Primer name | Primer Sequence (5' to 3') |
| --- | --- |
| *astA-F* | CTGCCGGTGTGATCGTTACT |
| *astA-R* | GAGCAGGGCTATGTGTTCGT |
| *astB-F* | GATGCACTTTGCCATCCAGC |
| *astB-R* | GCACGAGCGTCCGTTTATTC |
| *astC-F* | GCGGTCGTGAGCGAATTTAC |
| *astC-R* | GTTACACCAACGAGCCGGTA |
| *astD-F* | TTCGGCAAATGACCAGGGAA |
| *astD-R* | GCGAGCAGCGTAGTGAAATG |
| *astE-F* | TGTAGATCAAGGTGCCAGCG |
| *astE-R* | TGCTGAAAGCGGAGAAACCT |
| *16S rRNA-F* | CCTACGGGAGGCAGCAG |
| *16S rRNA-R* | ATTACCGCGGCTGCTGG |
| *fadR-F* | GGGTTTCGCGGAAGAGTACA |
| *fadR-R* | ATGGTCAACCAGCCATCTCG |
| *fadL-F* | TCCATCTGGTCGTAGCCTGA |
| *fadL-R* | TAGAAGCGCCCCAACCAAAT |
| *fadD-F* | GCAATATGCTGGCGAACCTG |
| *fadD-R* | AGGCAGTTAATGGTCAGGGC |
| *fadE-F* | CATCACCCTGCCTTCCAACT |
| *fadE-R* | AGCGGCTCTTCAATCCCTTC |
| *fadB-F* | GTCGTTAACCCTCGGCATGA |
| *fadB-R* | CGTTGGGTGGATTGTGGAGA |
| *fadA-F* | GCGGCCCTCGACGATATTTA |
| *fadA-R* | AGTGTGGTACTTCTGCCAGC |
| *acrA-F* | TACGCGCTATCTTCCCGAAC |
| *acrA-R* | GATCGGACGGGTTTCCACTT |
| *acrB-F* | ATCGCAGAGTTTAACGGCCA |
| *acrB-R* | CATAAACACGCCCTGGTCCT |
| *tolC-F* | AACTGGAAAGTGCCCATCGT |
| *tolC-R* | TTGAGCGGAAACTACGGCTT |
| *emrA-F* | TCACTCTCGACCCGACAGAT |
| *emrA-R* | GCGGTTGTAGTCGCTTTGTG |
| *emrB-F* | ATCATCCTTACCGTGGTGGC |
| *emrB-R* | GCAGAACAATAGCGCCGAAG |

Table S3 MIC values of FFC for different strains in this study

| Strains | MICs of FFC on strains (μg/ml) | Interpretation |
| --- | --- | --- |
| *S. suis* 11 | 64 | R |
| *S. suis* 089 yellow 2-1 | 64 | R |
| S*. aureus* ZYH 2-11 | 64 | R |
| S*. aureus* ZYH 2-3 | 64 | R |
| *Staphylococcus aureus* ZYH 1-2 | 64 | R |
| *K. pneumoniae* 090 blue | 16 | R |
| *K. pneumoniae* 1-20 C20 | 32 | R |
| *K. pneumoniae* 1-20 B23 | 32 | R |

MIC inflection point of FFC. *S. suis (*Using the CLSI breakpoints for *Streptococcus pneumoniae)*: S ≤ 2; I = 4; R ≥ 8. *S.* aureus: S ≤ 2; I = 4; R ≥ 8. K*. pneumoniae [*Refer to "Technical Specifications for Monitoring Antimicrobial Resistance in Animal-Derived *Klebsiella pneumoniae*" (DB 6501/T 045—2023)]: S ≤ 4; I = 8; R ≥ 16. S (Susceptible) indicates that the drug effectively inhibits microorganisms at the recommended dose; I, (Intermediate) indicates that the drug requires a higher-than-standard dose or specific conditions to be effective; R, (Resistant) indicates that due to the presence of resistance mechanisms, the drug cannot exert antimicrobial activity even at high concentrations.

Table S4 Minimum Inhibitory Concentrations (MIC) of Escherichia coli GQ95, FS83-1, and FS95 for Different Antibacterial Agents

| various drugs | Minimum Inhibitory Concentration (MIC) of *E. coli* GQ95 to Various Drugs (μg/ml) | Minimum Inhibitory Concentration (MIC) of *E. coli* FS 83-1 to various drugs (μg/ml) | Minimum Inhibitory Concentration (MIC) of *E. coli* FS95 to various drugs (μg/ml) | Interpretation |
| --- | --- | --- | --- | --- |
| Florfenicol | 32 | 16 | 32 | R |
| Ceftriaxone | 1024 | 1024 | 1024 | R |
| Oxacillin sodium | 1024 | 1024 | 1024 | R |
| Doxycycline | 16 | 32 | 64 | R |
| Levofloxacin | 4 | 64 | 8 | R |
| Ciprofloxacin | 8 | 64 | 16 | R |
| Ceftiofur sodium | 512 | 1024 | 512 | R |
| Amoxicillin | 1024 | 1024 | 1024 | R |

The allowable range of the minimum inhibitory concentration of different antibiotics for *E. coli* (μg/ml). Florfenicol: 2-8. Ceftriaxone: 1-4. Oxacillin sodium: 8-32. Doxycycline: 1–4. Levofloxacin: 0.25–1. Ciprofloxacin: 0.25–1. Ceftiofur sodium: 0.25–1. Amoxicillin: 2–8.

Table S5 FICI Values of E. coli GQ95, FS83-1, and FS95 for Different Antibacterial Agents

| various drugs | FICI of *E. coli* GQ95 to various drugs | FICI of *E. coli* FS 83-1 to various drugs | FICI of *E. coli* FS95 to various drugs | Interaction type |
| --- | --- | --- | --- | --- |
| Florfenicol | 0.28 | 0.25 | 0.16 | Synergism |
| Ceftriaxone | 0.375 | 0.31 | 0.31 | Synergism |
| Oxacillin sodium | 0.56 | 0.375 | 0.53 | Additivity |
| Doxycycline | 0.625 | 0.625 | 0.56 | Additivity |
| Levofloxacin | 0.75 | 0.75 | 0.75 | Additivity |
| Ciprofloxacin | 0.75 | 0.75 | 0.75 | Additivity |
| Ceftiofur sodium | 1.25 | 1.25 | 1.25 | Indifference |
| Amoxicillin | 1.5 | 1.5 | 1.25 | Indifference |

Note: FICI ≤ 0.5 is defined as synergistic effect; 0.5 < FICI ≤ 1 is defined as additive effect; 1 < FICI ≤ 4 is defined as no effect; FICI > 4 is defined as antagonistic effect.


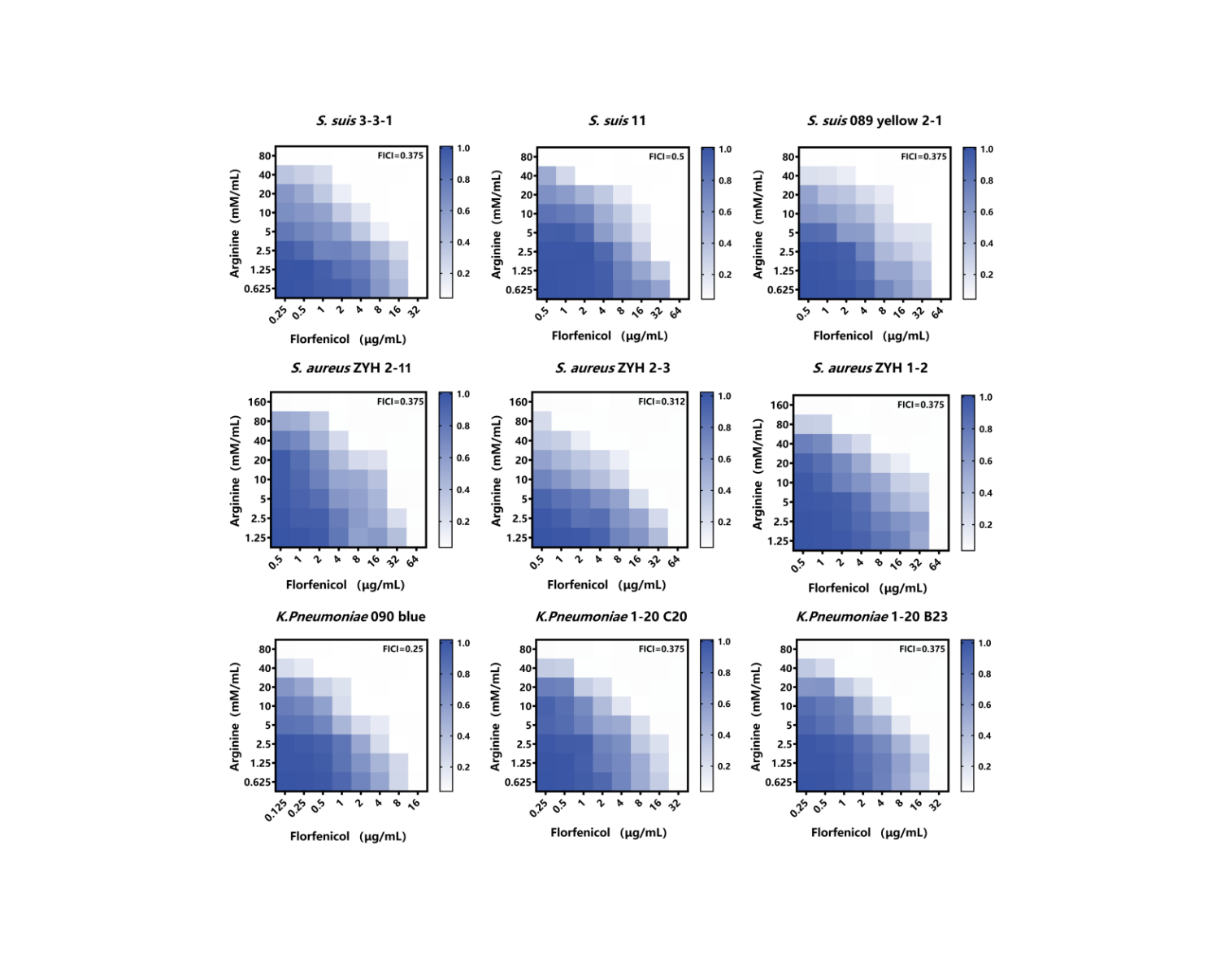


Figure S1. Synergistic effects of Arg and FFC against various clinical isolates (synergy defined as fractional inhibitory concentration index (FICI) ≤ 0.5).


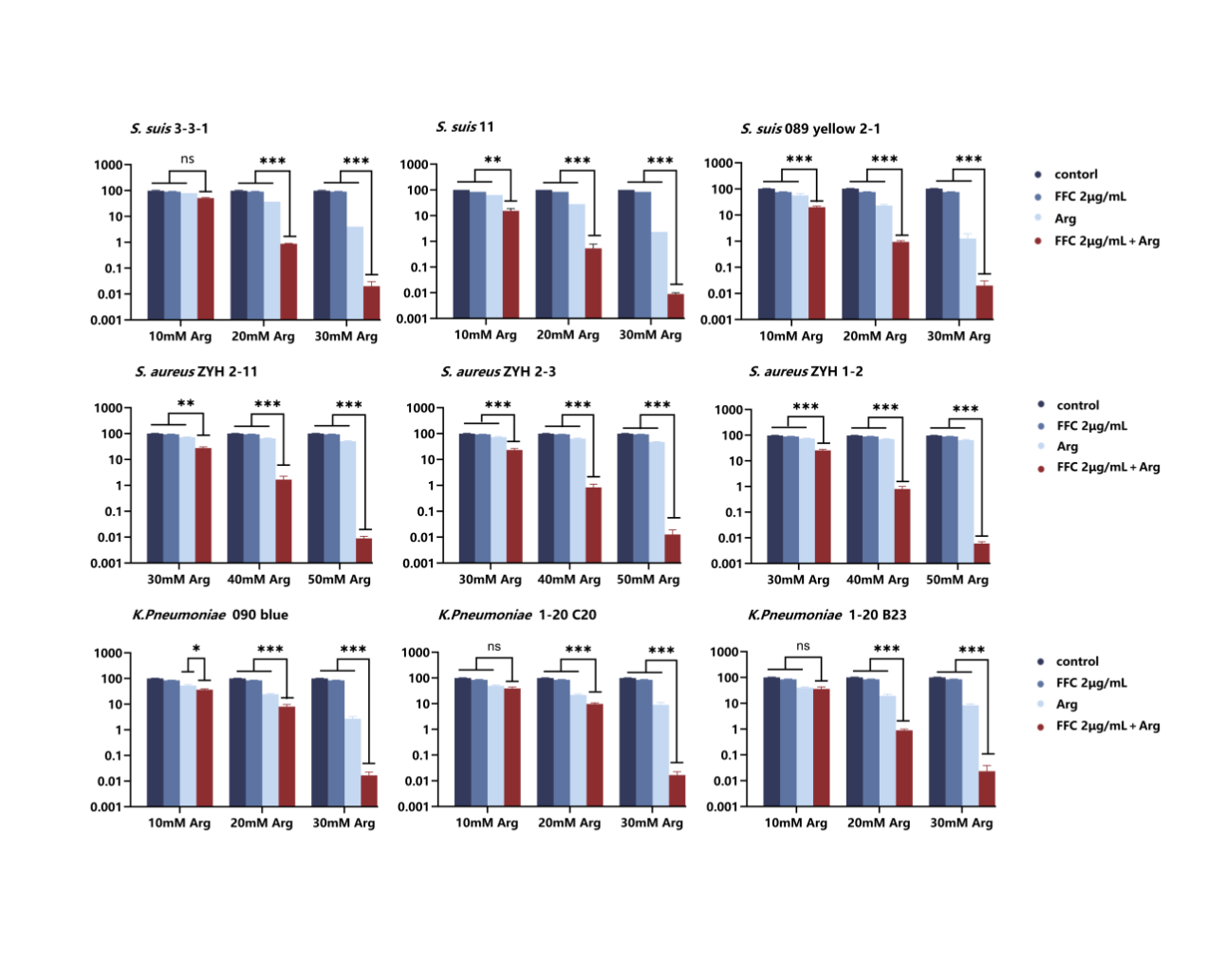


Figure S2. Effect of Arg at different concentrations combined with 2 μg/mL FFC on survival rates of various clinical isolates.


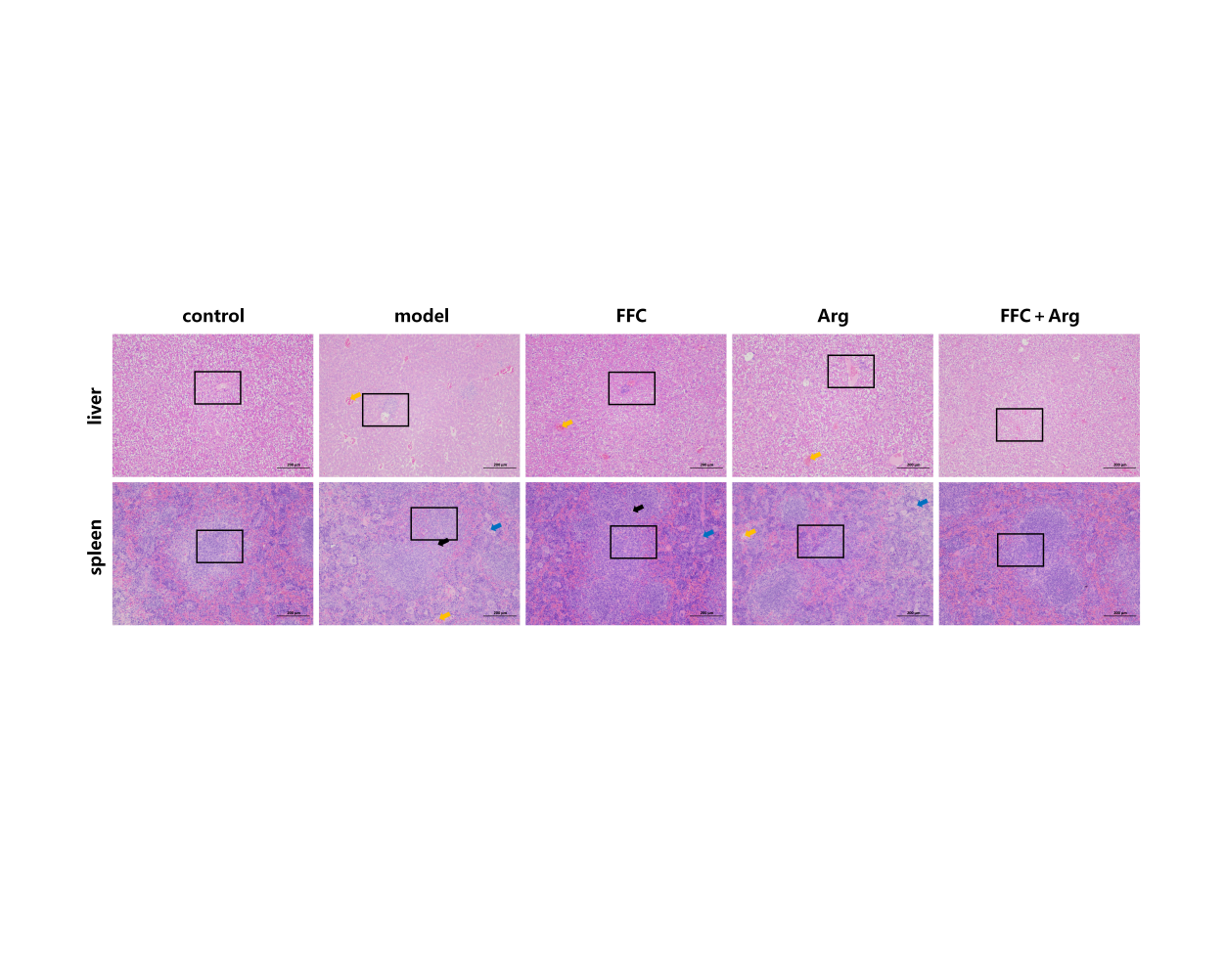


Figure S3. H&E staining results of liver and spleen in a mouse bacterial peritonitis infection model. Liver: vascular congestion (orange arrow); Spleen: fusion of white pulp (black arrow), megakaryocytosis (blue arrow), congested and dilated sinusoids (orange arrow).


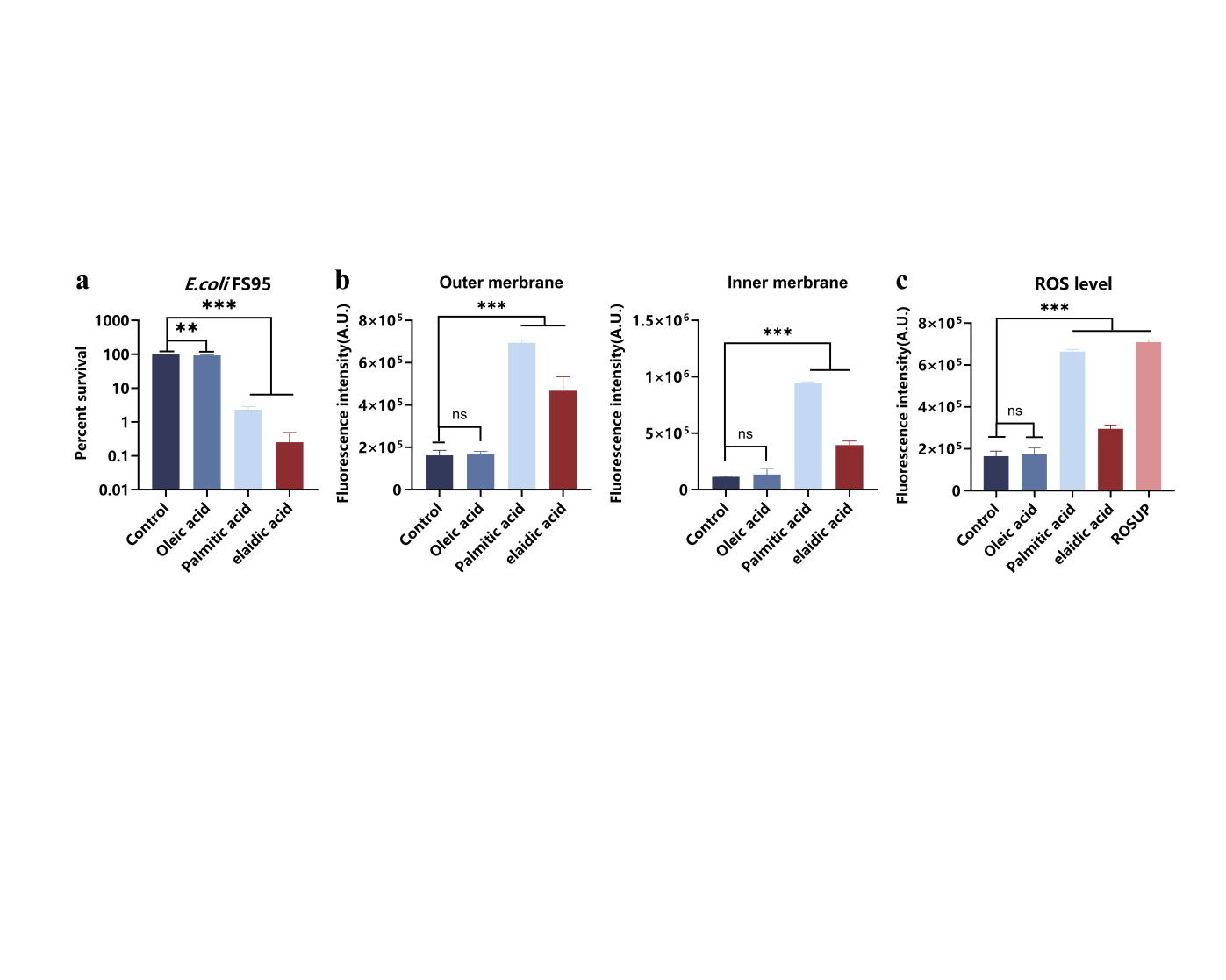


Figure S4. Exogenous addition of free fatty acids (FFAs) reproduces the core phenotype of combined drug treatment and induces bacterial death. a) Effect of exogenous FFAs on bacterial survival rate; b) Effect of exogenous FFAs on cell membrane integrity; c) Effect of exogenous FFAs on intracellular ROS levels.


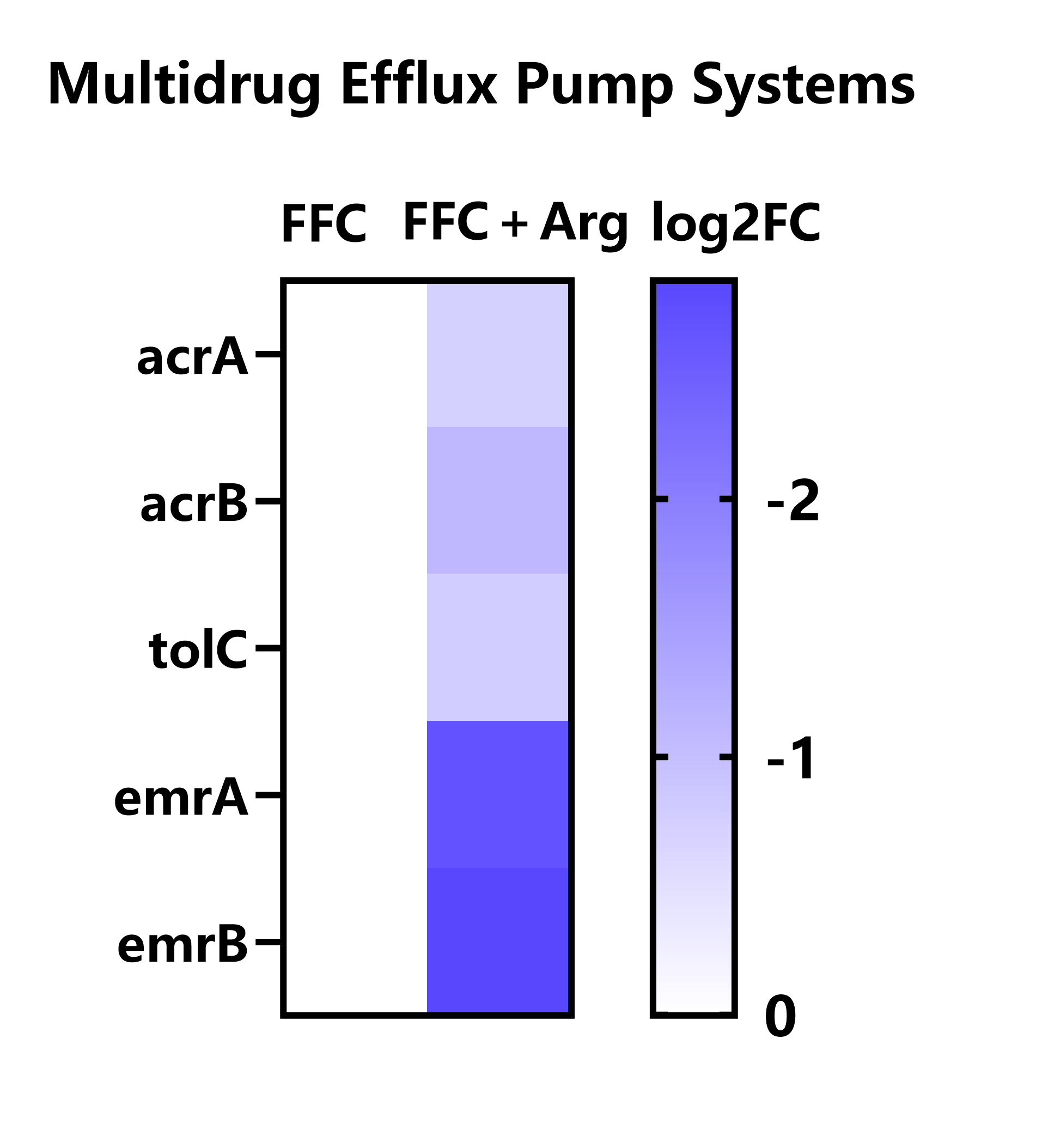


Figure S5. Heatmap of differentially expressed efflux pump proteins.
